# Control of Type 1 and Type 2 Errors in Configural Frequency Analysis

**DOI:** 10.17505/jpor.2026.29048

**Published:** 2026-03-26

**Authors:** Stefan von Weber, Alexander von Eye

**Affiliations:** 1 Universität Furtwangen, Germany; 2 Michigan State University, USA

**Keywords:** Configural Frequency Analysis, CFA, Victor CFA, type strength, Type I error, Type II error

## Abstract

In this article, standard configural frequency analysis (CFA) is compared with combinatorial Victor CFA with respect to Type I errors and power. Victor CFA is most important and suitable when phantom types or phantom antitypes are suspected to exist. These can emerge when types and antitypes contain cases from populations other than the sample under study, and, thus, distort marginal probabilities. Simulation results are presented that suggest that, when samples are comparatively small, standard CFA has more power. In contrast, Victor CFA has more power when sample sizes increase. The concept of type strength is introduced. It can be used as a supplement to statistical CFA testing. Data examples illustrate that the two variants of CFA can result in dramatically different data evaluations. In the article, it is discussed that Victor CFA can also be used when base models are more complex than the main effect model that was used in Lienert’s standard CFA and Victor’s original alternative CFA.

## Introduction

Person-oriented research proceeds from the assumption that generalization of results from samples to populations can be too crude to properly represent each individual (see Bergman, & Magnusson, 1997; Lundh, 2023; von Eye, & Bergman, 2003; von Eye, Bergman & Hsieh, 2015). Therefore, homogenous subgroups or even individuals are either a priori defined or searched for. In searches for homogeneous groups of individuals, Configural Frequency Analysis (CFA; Lienert, 1968; Krauth, & Lienert, 1973; Lautsch, & von Weber, 1995; Lienert, & Krauth, 1975; von Eye, & Gutiérrez Peña, 2004; von Eye, & Wiedermann, 2021) plays an important role. CFA allows researchers to define homogeneity based on hypotheses that concern effects that might exist in cross-classifications. Subgroups contradict a priori specified hypotheses in particular ways.

The search for such subgroups proceeds by way of comparison of observed cell frequencies with those frequencies that are expected under the assumptions of some base model. CFA identifies significantly over- or under-frequented cells. Over-frequented cells are said to constitute CFA types. Under-frequented cells are said to constitute CFA antitypes.

The hypotheses under which a CFA is performed are reflected in the CFA base model (von Eye, 2004; von Eye, & Wiedermann, 2021). The base model contains all effects the researchers are not interested in. When this model is rejected, the effects of interest are bound to exist. In exploratory CFA, each cell of a cross-classification is tested. This involves multiple tests. Therefore, a suitable method of protection of the local *α_L_* is applied to maintain the global Type I error rate, *α_G_*. Methods of protection include, for example, the ones proposed by Bonferroni (1936) or Holm (1979). Because the local level *α_L_* is protected, the number of identified extreme cells usually is reduced. In addition, there is a complication – the possible occurrence of so-called *Victor types* (constituted by cells that contain more cases than expected) and *Victor antitypes* (constituted by cells that contain fewer cells than expected).

Victor (1983) and Kieser and Victor (1999) were the first to introduce the concept of *structural zero* into the methodology of CFA. A structural zero is a cell in the contingency table where, for logical or subject-specific reasons, only a frequency of zero can occur. The authors applied the concept to type cells, that is, cells whose frequency is far above the frequency calculated from the product of the marginal probabilities (*Victor type*).

In simulations of contingency tables for CFA, we included tables with Victor types. For this, we have introduced the type strength *τ* (von Weber, & von Eye, 2005). Let the expected cell frequency, *ê_i_*, be the estimated expected value for Cell *i*, and *n_i_* the corresponding observed frequency, then type strength is given by the quotient

$$\tau =(n_{i}/e_{i})-1.$$

A cell in which the frequency *n_i_* and the expected value *ê_i_* are the same has a type strength of *τ* = 0. For antitypes, that is, for cells where *n_i_* < *ê_i_*, the type strength becomes negative, but can only take the value -1 as a minimum because *n_i_* ≥ 0. Whether a type begins to distort a standard CFA and, thereby, becomes a Victor type depends on the type strength *τ*, but also on other factors and table properties.

In the present article, we do not discuss the field-specific justification for the existence of Victor types. Instead, we focus on the statistical errors that generally can occur during analysis. Victor and Kieser have proposed combinatorial search for the evaluation of tables that might contain types. The main reason for using this variant of CFA is that a Victor type, due to its excessively high frequency, distorts the estimation of marginal probabilities to such an extent that there is an increased occurrence of *phantom types* that may have no statistical or field-specific relevance.

von Weber, von Eye, and Lautsch (1995) had already programmed, and successfully applied, the combinatorial search, at that time in the programming language Pascal. They arrived at the same results as Victor and Kieser, who used loglinear modeling. In the present article, we present the results of a re-implementation of the combinatorial type search within an extended CFA methodology, this time as a C program. The reason is the enormous computational power of C programs, which is nearly as high as that of a program written in machine language. The new implementation allows for simulations to be conducted alongside the usual table analysis, with which the expected Type I and Type II errors can simultaneously be estimated.

This article is structured as follows. First, we describe the combinatorial algorithm that will be used in the search for types and antitypes. Second, we present the methods used for the generation of cross-classifications in the simulations. Third, we resume the discussion of the combinatorial search for types and antitypes. Specifically, we present this method within the context of a general CFA model. In this context, the steps of the simulation are presented. Simulations are performed with the goal of comparing standard CFA with Victor and Kieser’s CFA. This is followed by a discussion of simulation results. Issues of α protection are discussed next. A second simulation is presented in which the sensitivity to methods of α protection in the detection of types and antitypes under the two versions of CFA is examined. Data examples are presented before a general discussion.

## The Pure Combinatorial Search Algorithm

In combinatorial search, a subset of the cells in a table is tentatively treated as Victor types. Then, in the first step, the marginal probabilities of the table are estimated by setting the frequencies of the Victor type cells to zero. Using the Deming-Stephan algorithm, approximative values are calculated for the expected values of all cells, including the Victor type cells, under the assumed base model. This is an iterative process. After about 20 iterations, estimates usually change no longer. It is the purpose of this methodology to obtain a better estimate from the base model, that is, cell probabilities without the distortion that possibly is caused by Victor types or structural zeros.

Given is a contingency table { *n_i_* } with *N* cells (or symptom configurations) where *n_i_* is the observed frequency of cases with symptom configuration (*i*). The dimension of the table is *d* ≥ 2. Kieser and Victor assume that most of the cells obey the base model of variable independence, and only a few type cells or antitype cells are outliers (see also von Eye, & Wiedermann, 2025). It can be shown, however, that the concept of Kieser types can be applied to any CFA base model (given sufficient degrees of freedom).

In the simulations presented in this article, we proceeded in the following steps.

*Step 1*: Estimation of the maximum number *n_t,max_* of types to search for. This number is calculated using the formula *n_t,max_* = INT[df ^0.5^+0.01] where *df* is the degree of freedom of the table. For example, for *df* of 4 ≤ *df* ≤ 8 we find *n_t,max_* = 2. Then, a first Cell of the table is marked, and the number *n_t_* of actually assumed type cells is set to *n_t_* = 1.

*Step 2*: Calculation of the so-called Victor expectancies *ê_i_* of all cells using the Deming-Stephan algorithm. Marked cells are assumed to be type cells or antitype cells. This is followed by calculation of the two chi-squared sums, the first from all marked cells, the second from all non-marked cells:

$$\chi_{\text{t}}^{2}=\sum_{marked}\frac{{\left(n_{i}-e_{i}\right)}^{2}}{e_{i}}$$ (1)

$$\chi_{\text{non}}^{2}=\sum_{unmarked}\frac{{\left(n_{i}-e_{i}\right)}^{2}}{e_{i}}$$ (2)

With χ^2^_t_ and χ^2^_non_, we calculate the F-statistic

$$F=\frac{\chi_{t}^{2}\left(N_{c}-n_{t}\right)}{\chi_{non}^{2}\cdot n_{t}}$$ (3)

*Step 3*: Mark the next cell and repeat Step 2. Continue until all single cells were tested. If *n_t,max_* >1 holds, continue with pairwise combinations of marked cells (case *n_t_* =2), e.g., (1,2), (1,3), … When *n_t,max_* >2 holds, continue with triples of marked cells (case *n_t_* =3), e.g., (1,2,3), (1,2,4),,…. This combinatorial search is continued until *n_t_* = *n_t,max_*. From all calculated F-values, we take the maximum F*_max_* and store the vector of the corresponding Victor expectancies, ê^*^_i_, but not the combination of type cells.

*Step 4:* The confirmatory hypothesis tests are performed with the local cell test of Dunkl and von Eye (1990) using the Victor expectancies ê*_i_ from Step 3. α protection can be done with Bonferroni’s adjustment. The test statistic is

$$X=\frac{n_{\text{i}}-{\hat{e}}_{\text{i}}}{\sqrt{\sigma_{\text{i}}^{2*}}},$$

with variance σ²*_i_ = *ê_i_* (*ê_i_* + 0.5)/(*ê_i_* – 0.5), and continuity correction as introduced by Dunkl and von Eye (1990).

## Generation of Random Tables with Types and Antitypes

In the simulation of contingency tables, we determine the number *n_t,max_* of the set type cells, their position in the table, and the type strengths. The number of set types is based on the above formula, that is, *n_t,max_ =* INT *[df ^0.5^+0.01]*. We use 2 × 2 × 3 × 2 tables. These tables have, under the base model of variable independence, *df* = 19, and *n_t,max_* was set to 4. The positions of the type cells were fixed in all simulations. Specifically, in tables spanned by 4 variables and 24 cells, Cells 4, 12, and 19 contained the 3 types, and Cell 22 contained the sole antitype. Type strength varied for the types from τ = 0.5 to τ = 3.0 in steps of 0.5, while the strength of the antitype varied between -1/3 and -3/4. The recalculation of an antitype strength *τ_antitype_* into a comparable type strength τ is achieved using the formula *τ=τantitype*/1+τ*antitype*. Thus, τantitype = -3/4 corresponds to the type strength τ = 3. The upper limit of antitype strength was set to -3/4 because, for antitype strengths close to the value of -1, implausibly high comparable type strengths τ arise.

An important characteristic of a contingency table is the mean cell frequency n_ijk_, as tables with a high mean frequency generally provide more accurate results than those with a low mean frequency. Therefore, in our simulations, values of n_ijk_=10 and n_ijk_=20 were used.

The total number of combinations *n_combs_* to be calculated of assumed type cells for a table with ZZ = 24 cells and a maximum of *n_t,max_*= 4 positions of type cells to be combined is

$$n_{combs}=\sum_{j=1}^{4}\left(\begin{array}{c} ZZ\\ j \end{array}\right)=12,950.$$

For each table, all combinations with one type cell, with two type cells, with three, and with four type cells are created. For each of these, the Deming-Stephan algorithm calculates new Victor expectation values ê*_ijkl_. However, the actual analysis, that is, the identification of types/antitypes according to the small group test of Dunkl and von Eye (1990), is performed only five times for the table. The first analysis is performed for the original table with expected values from standard CFA. The next four analyses are then performed with Victor expected values for one, two, three, and four preset types, always for the combination with the highest *F*-value.

In each run, 200 tables were simulated, resulting in a computing time of just over an hour. The results were recorded in a 3-dimensional table. One dimension of the table consists of cells 1 - 24, the second dimension represents the distinction between type and antitype, and the third dimension represents the number *j* of set and combined type cells with *j* = 0, 1, 2, 3, 4.

The creation of a simulated table is performed in the following seven steps:

(1) Generation of the design matrix;
(2) Generation of the marginal proto-probabilities (these are the probabilities used in the simulations);
(3) Calculation of the cell-wise proto-probabilities and normalization to sum 1;
(4) Calculation of the proto-frequencies (these are the frequencies used in the simulations);
(5) Multiplication of the proto-frequencies of the type cells with the type strength;
(6) Addition of random, normally distributed fluctuations to proto-frequencies;
(7) Summation of frequencies and normalization of the sum to the mean frequency n*_ijkl_*.

In more detail, the seven steps are:

(1) Generation of the design matrix: In a 2 × 2 × 3 × 2 table, there are four variables with the manifestations (0, 1), (0, 1), (0, 1, 2), (0, 1) (see sample table below).
(2) Generation of proto marginal probabilities: To generate the preliminary marginal probabilities, we use the C function rand() and the formula 0.2 + 1.0*rand()/RAND_MAX. The expression 1.0*rand()/ RAND_MAX generates a random number within the interval [0, 1], whereby the limit 1 itself is not reached. Adding 0.2 corresponds to invoking the Delta option and prevents small products from being generated too often when multiplying marginal probabilities.
(3) Calculation of proto-probabilities for each cell and normalization to sum 1: The product of the marginal proto-probabilities for a cell yields a cell protoprobability. The sum of all cell proto-probabilites is calculated and normalized to 1. This determines the proto-cell probabilities.
(4) Calculation of proto-frequencies: By multiplying the cell proto-probabilities with the given mean frequency and multiplying with the number of cells, 24, we obtain proto-frequencies, *n’_ijkl_*, that correspond to the base model, that is, do not yet have any deviations from it.
(5) Multiplication of the proto-frequencies of the type cells by their corresponding type strength: The three a priori determined type cells (4, 12, and 19) are multiplied by the value τ + 1, and antitype cell 22 is multiplied by the value τ_antitype_+1. This results three types and one antitype. We now have a modified proto-frequency vector $n_{ijkl}^{"}$ which contains defined deviations from the base model.
(6) Addition of random, normally distributed fluctuations to the proto-frequencies: Frequencies *n_ijkl_* are asymptotically affected by variance σ^2^ = *n_ijkl_*. We generate N(0;1)-distributed random numbers by adding 12 uniformly distributed random numbers from the interval [0, 1] and then subtracting the value 6 from the sum. We then introduce randomness by adding
  $$N\left(0;1\right)\sqrt{n_{ijkl}^{"}}$$
  to each proto-frequency from step (5). When negative numbers arise, they are set to 0. We now have a modified proto-frequency vector $n_{ijkl}^{\prime \;\prime \;\prime }$ with random fluctuations.
(7) Summation of the proto-frequencies and normalization of the sum to the mean *n_ijkl_* : The addition of the type cells can significantly alter the mean of the frequencies, and so can random fluctuations. Therefore, all proto-frequencies $n_{ijkl}^{\prime \;\prime \;\prime }$ are summed, to obtain $\sum \;\;n_{ijkl}^{\prime \;\prime \;\prime }$. Subsequently, all frequencies are multiplied by the normalization factor ${\bar{n}}_{ijkl\;}ZZ/\sum \;n_{ijkl}^{\prime \;\prime \;\prime }$ and, then, the sum of the final frequencies *n_ijkl_*, i.e., *N_t_=∑*n_ijkl_, is normalized, to obtain the target mean, *n_ijkl_*. The generated table is now ready for analysis. (For programming reasons, the simulation program treats the frequencies up to and including step (7) as fractional numbers. Therefore, a slight difference from the desired target mean may occur after rounding to whole numbers.)

### Combinatorial Search for Types and Antitypes

The search involves the following two steps of analysis:

(1) Standard CFA without calculating Victor expectations
(2) Combinatorial search with 1, 2, …, *n_t, max_* suspected type cells, based on Victor expectations.

More specifically, the two steps are:

(1) Standard CFA without calculating Victor expectations. As usual, the expectations are estimated using the standard CFA base model. This model can, in the present context, be represented by the log-linear model
  $$\log\hat{m}=X\lambda ,$$
  where $\hat{m}$ is the vector of estimated expected cell frequencies, *X* is the design matrix that represents the effects taken into account, that is, main effects, interactions, and covariates, and λ is the parameter vector (rules for setting up CFA base models are given in von Eye, 2004, and in von Eye, Wiedermann, and von Weber, 2021; a more general rendering of the general CFA base model is given in von Eye, and Wiedermann, 2025). In standard CFA and without taking into account structural zeros, the expected cell frequencies can be estimated from the marginals. In the present simulations, we consider the model of variable independence, and tested for possible types or antitypes using the local cell test of Dunkl and von Eye (1990). The log-linear model is, thus, a main effect model. We adjust α according to Bonferroni, i.e., α*= α / *ZZ* (here with the number of cells, *ZZ* = 24).
(2) Combinatorial search with *j* = 1, 2, .., *n_t,max_* presumed type cells using Victor expectations: when only one type (*j* = 1) is hypothesized, cells 1 - 24 are successively assumed to be type cells. Victor expectations are calculated under this assumption, their sum is normalized to the value *N_t_*, and the *F* - value is calculated. The combination (here a combination is only one cell) with the highest *F* - value is analyzed, that is, it is tested for types and/or antitypes. The CFA base model for this part of the analysis is
  $$\log\hat{m}=X\left(\begin{array}{c} \lambda \\ \tau \end{array}\right),$$
  where, as in the first step of the simulation, $\hat{m}$ is the vector of estimated expected cell frequencies, *X* is the design matrix, and λ is the parameter vector. Here, however, *X* consists of two parts. The first is the same as for standard CFA. That is, it represents the effects taken into account in standard CFA, that is, main effects, interactions, and covariates. λ contains the parameters for this part of the analysis. The second part of *X* contains the vectors that identify cells as structural zeros. There is one vector per structural zero. The parameter vector is, thus, augmented by τ, that is, the parameters that represent the columns in the second part of *X*. Without the second part of *X* and without *τ*, this model is equivalent to the one used for standard CFA.

For two suspected types (*j* = 2), all combinations of two cell numbers, that is, (1, 2), (1, 3), …, (23, 24), are tested. For each combination (here, a combination of two cell numbers), the Victor expectations and the F-value are calculated. Here, too, only the combination with the highest F-value is tested for the presence of types and/or antitypes.

For three suspected types (*j* = 3), all combinations of 3 cell numbers are analyzed, that is, (1, 2, 3), (1, 2, 4), …, (22, 23, 24). For four suspected types, all combinations of 4 cell numbers are played through, that is, (1, 2, 3, 4), (1, 2, 3, 5), …, (21, 22, 23, 24), etc. An example of the tables created for the following simulation appears in Table 1.

**Table 1:** Example of a simulation table with type strengths τ = 2.5 for Cells 4, 12, 19 and antitype strength τantitype = -0.71 for Cell 22, and Nt = 231, mean frequency n_ijkl_ = 9.625 and local αL=0.05, Bonferroni adjustment, and j = 4 assumed type cells

Cell number	I	J	K_1_	K_2_	L	*n_ijkl_*	*E_ijkl_*	*V_ijkl_*	TV	CEP	*t*	T/A
1	0	0	1	0	0	8	8.05	6.22	0.74	0.46	0	
2	0	0	1	0	1	6	11.50	9.22	-0.92	0.36	0	
3	0	0	0	1	0	7	4.67	4.72	1.04	0.30	0	
4	0	0	0	1	1	15	6.67	6.99	3.11	1.910-3	1	Type
5	0	0	-1	-1	0	4	6.13	3.76	0.25	0.80	0	
6	0	0	-1	-1	1	6	8.76	5.57	0.31	0.76	0	
7	0	1	1	0	0	4	13.79	8.17	-1.36	0.17	0	
8	0	1	1	0	1	13	19.71	12.11	0.26	0.79	0	
9	0	1	0	1	0	9	7.99	6.20	1.10	0.27	0	
10	0	1	0	1	1	6	11.43	9.19	-0.69	0.49	0	
11	0	1	-1	-1	0	4	10.50	4.94	-0.20	0.84	0	
12	0	1	-1	-1	1	38	15.01	7.32	10.96	1.010-6	1	Type
13	1	0	1	0	0	5	7.50	7.36	-0.59	0.56	0	
14	1	0	1	0	1	12	10.72	10.90	0.61	0.55	0	
15	1	0	0	1	0	4	4.35	5.58	-0.30	0.77	0	
16	1	0	0	1	1	8	6.22	8.27	-0.08	0.94	0	
17	1	0	-1	-1	0	4	5.72	4.45	0.11	0.91	0	
18	1	0	-1	-1	1	4	8.17	6.59	-0.92	0.36	0	
19	1	1	1	0	0	34	12.85	9.66	7.50	1.010-6	1	Type
20	1	1	1	0	1	18	18.37	14.31	0.96	0.34	0	
21	1	1	0	1	0	5	7.45	7.33	-0.78	0.44	0	
22	1	1	0	1	1	2	10.65	10.86	-2.48	0.013	1	
23	1	1	-1	-1	0	6	9.79	5.84	0.40	0.69	0	
24	1	1	-1	-1	1	9	14.00	8.65	0.12	0.91	0	

Here, *I, J, K, L* represent the symptoms, *n_ijk_* the frequencies, *E_ijk_* the usual expectations, estimated, as in standard CFA from a log-linear main effect base model. *V_ijk_* are the Victor expectations (estimated, using the above formula, for the 4-combination marked in column *t*), *TV* are the test values (these are *z*-scores), *CEP* the critical error probability, *t* the column of the cell combination of the suspected type cells (here, a 4-combination), and column T/A with the result whether a type or an antitype was identified.

The results in Table 1 suggest that combinatorial search in this example was able to identify each of the type cells (4, 12, 19, and 22). However, the subsequent tests could only verify the types in cells 4, 12, and 19. Although the antitype cell 22 was correctly included in the four-type combination, it was not verified as an antitype because the test value of *TV* = -2.48 is too small in magnitude, that is, the critical error probability of *CEP* = 0.0075 exceeds the Bonferroni-protected α*=0.05/24 = 0.00208.

## Results of the Simulation

The global Type I error, *α_G_*, is the global probability for the entire table that cells are mistakenly tested as type or antitype. The Type II error (*β*) is the probability that set types/antitypes are not detected. *Power = 1 - β* is the probability that a set type or antitype is detected.

To estimate *α_G_*, the number *n_f_* of falsely tested types/antitypes is calculated for cells 1 - 3, 5 - 11, 13 - 18, 20, 21, 23, and 24, that is, the frequencies in all cells except the 4 cells set, that is, 4, 12, 19, and 22. This summation is performed over all 200 tables of a program run. Then, the estimate of *α* is *α_G_* = *n_f_* / 200.

The test power 1 *- β* varies considerably for types and antitypes. Types are considerably easier to verify than antitypes (cf., Indurkhya, & von Eye, 2000). Therefore, the results are presented separately for types and antitypes. To estimate the type II error (*β*) or the *Power* = 1 - β of types, the number *n_tt_* of tested types is summed for cells 4, 12, and 19, across all 200 tables of a program run, that is, a total of 600 types. Then, the power estimate for these types is *Power_t_ =* 1 - β*_t_ = n_tt_* / 600.

For the test power of antitypes, the number *n_tAt_* of tested antitypes is summed for cell 22, across all 200 tables of a program run, that is, a total of 200 antitypes. Then, the power estimate for the antitypes is *Power_tAt_ =* 1 - β*_tAt_ = n_tAt_* / 200.

In the simulations, the two parameters *average frequency n*_ijkl_ and *type strength τ* were varied. The average frequency had the two values *n*_ijkl_ = 10 and *n*_ijkl_ = 20, and the type strength had the values τ = 0.5, 1.0, 1.5, 2.0, 2.5, and 3.0, resulting in 2 × 6 = 12 program runs. For each of the 200 simulated tables, five search runs were performed: (j = 0), that is, one run without Victor expectations (standard CFA), and (j = 1 - 4), that is, for runs with Victor expectations and cell combinations of j = 1, 2, 3, and 4 cells. Table 2 summarizes results.

**Table 2:** Global error of the first type (αG) and power (1-β) for the detection of types under variation of mean frequency, n_ijkl_, and type strength, τ

*n*_ijk_	τ	*α_G_* 1-β	j=0	j=1	j=2	j=3	j=4
10	0.5	*αG*	0	0.04	0.18	0.35	0.68
		1-β	0.02	0.10	0.13	0.16	0.17
10	1	*αG*	0.01	0.02	0.16	0.33	0.57
		1-β	0.09	0.22	0.28	0.30	0.31
10	1.5	*αG*	0.01	0	0.07	0.24	0.50
		1-β	0.31	0.29	0.38	0.43	0.43
10	2	*αG*	0.01	0.01	0.15	0.34	0.54
		1-β	0.48	0.40	0.43	0.48	0.50
10	2.5	*αG*	0.04	0.01	0.13	0.26	0.51
		1-β	0.60	0.47	0.48	0.56	0.57
10	3	*αG*	0.05	0.02	0.16	0.30	0.51
		1-β	0.65	0.54	0.51	0.58	0.59
20	0.5	*αG*	0.02	0.05	0.21	0.43	0.76
		1-β	0.04	0.16	0.19	0.22	0.26
20	1	*αG*	0.04	0.01	0.19	0.46	0.74
		1-β	0.37	0.34	0.41	0.45	0.46
20	1.5	*αG*	0.05	0.01	0.09	0.30	0.48
		1-β	0.56	0.46	0.51	0.57	0.59
20	2	*αG*	0.20	0.07	0.09	0.14	0.28
		1-β	0.75	0.66	0.62	0.68	0.72
20	2.5	*αG*	0.17	0.13	0.10	0.18	0.19
		1-β	0.81	0.72	0.69	0.70	0.75
20	3	*αG*	0.48	0.19	0.17	0.09	0.20
		1-β	0.86	0.78	0.75	0.76	0.80

The shaded fields indicate maximum power with the best possible compliance with the global type I error (α_G_). As can be seen, for tables with mean frequency *n*_ijkl_ = 20 and type strength τ ≥ 2.0, specifying a local α_L_ = 0.05 is no longer sufficient to guarantee a global α_G_ ≤ 0.05, and that despite Bonferroni protection.

Tables with *mean frequency n*_ijkl_ = 10 and type strength τ <1.5 have a very poor power of 20%, or less. For tables with *mean frequency n*_ijkl_ = 10 and *type strength τ* between 1.5 and 3.0, standard CFA is clearly the method of choice. While maintaining a global *α_G_* = 0.05, it yields the highest power, with values between 30% and 65%. The same applies to tables with *mean frequency n*_ijkl_ = 20 and type strength τ between 1.0 and 1.5.

For tables with *mean frequency n*_ijkl_ = 20 and *type strength* τ ≥ 2.0, the combinatorial search is the better choice. However, the difficulty here is finding the correct specification of the local *α_L_* and the correct number *j* of combinations in the combinatorial search. For a table with observed data, one only knows the *mean frequency n*_ijkl_, but not the strengths of the types hidden in the numbers. Therefore, in the next section, we attempt to develop criteria that help in the search for the optimal local *α_L,_* and the correct number *j* of combinations.

As was indicated above, antitypes are hard to detect. Among the main reasons for this is that cell frequencies cannot be smaller than zero. To give an example, the type strength of *τ* = 2.0 corresponds to a multiplication factor of *τ +* 1 =3.0. That is, to obtain the simulated observed frequency, the expected frequency, *e*_ijkl_, is multiplied by 3.0. A type strength of *τ* = 2.0 corresponds to an antitype strength of $\tau_{\text{antitype}}=-\frac{\tau }{\tau +1}=‐2/3$, and the multiplication factor becomes *τ+1* = 1/3. That is, to obtain the simulated observed frequency, the expected frequency, *e*_ijkl_, is multiplied by 0.33. For an average cell frequency of *n*_ijkl_=10 and an expected cell frequency of *n_ijkl_*=10, the cell frequency for a type of *τ* =2.0 increases from *n_ijkl_* = 10 to *n_ijkl_* = 30. For an antitype, this frequency is reduced from *n_ijkl_* = 10 to *n_ijkl_* = 3. The test statistics - they contain, as their central elements, the quotient (*n_ijkl_* - *e*_ijkl_) / *e*_ijkl_ - are, then, for the type, (30 - 10)/10 = 2.0, and, for the antitype, (3 - 10)/10 = - 0.7, a much smaller absolute value.

From Table 3, it can be concluded that the search for antitypes in sparse tables (*n*_ijkl_=10) is better conducted using the combinatorial approach, in particular when the local significance threshold is lowered, that is, *α_L_* < 0.05. When tables contain more cases (*n*_ijkl_=20), standard CFA is promising as well. Again, data analysts may wish to consider lowering the local significance threshold.

**Table 3:** Global error of the first type (αG) and power (1-β) for the detection of types under variation of mean frequency, n_ijkl_, and type strength, τ

*n*_ijk_	τ	*α_G_* 1-β	j=0	j=1	j=2	j=3	j=4
10	**0.5**	*α_G_*	0	0.04	0.18	0.35	0.68
		1-β	0.00	0.01	0.02	0.02	0.02
10	**1.0**	*α_G_*	0.00	0.02	0.16	0.33	0.57
		1-β	0	0.01	0.02	0.03	0.02
10	**1.5**	*α_G_*	0.01	0	0.07	0.24	0.50
		1-β	0.01	0.01	0.01	0.03	0.02
10	**2.0**	*α_G_*	0.01	0.01	0.15	0.34	0.54
		1-β	0.02	0.01	0	0.03	0.03
10	**2.5**	*α_G_*	0.04	0.01	0.13	0.26	0.51
		1-β	0.04	0	0.01	0.03	0.09
10	**3.0**	*α_G_*	0.05	0.02	0.16	0.30	0.51
		1-β	0.05	0.02	0.02	0.03	0.06
20	**0.5**	*α_G_*	0.02	0.05	0.21	0.43	0.76
		1-β	0.02	0.01	0.01	0.03	0.04
20	**1.0**	*α_G_*	0.04	0.01	0.19	0.46	0.74
		1-β	0.06	0.13	0.14	0.17	0.14
20	**1.5**	*α_G_*	0.05	0.01	0.09	0.30	0.48
		1-β	0.15	0.11	0.18	0.16	0.18
20	**2.0**	*α_G_*	0.20	0.07	0.09	0.14	0.28
		1-β	0.29	0.18	0.11	0.16	0.25
20	**2.5**	*α_G_*	0.17	0.13	0.10	0.18	0.19
		1-β	0.39	0.18	0.09	0.09	0.24
20	**3.0**	*α_G_*	0.48	0.19	0.17	0.09	0.20
		1-β	0.49	0.28	0.16	0.13	0.29

## Further Protection of the Local Level *α_L_*

Tables 2 and 3 suggest that it is possible to enforce a threshold for the α error, e.g., *α_G_* =0.05, when the local level, *α_L_*, is protected even more than by the Bonferroni (or similar) procedure. More strict protection, however, comes at the price of losses in power.

For additional protection, we introduce the factor *f_k_* = 0.95*^k^*. The value of 0.95 was chosen arbitrarily. It serves as the base for a stepwise reduction of *f_k_* for increasing *k*. For *k* = 0, we set *f_k_* = 1. For *k*=10, one obtains *f_k_* = 0.95^10^ ~ 0.6, and for *k* = 100, *f_k_* = 0.95^100^ ~ 0.006. To obtain an overview of the band width of the values of parameter *k*, we varied such table characteristics as average frequency, *n*_ijkl_, average type strength, τ, and the number *j* of the combinatorial Victor type cells, with *j* = 0, 1, 2, …

The average frequency, *n*_ijk_, can easily be calculated from a given frequency table. It is *n*_ijk_=∑*n_ijk_ / ZZ*. Average type strength can be estimated by, first, conducting a standard or a Victor CFA and estimating the type strength for the identified type cells based on the observed cell frequencies and their corresponding expected frequencies, *e*_ijkl_ (or *V_ijkl_*). In standard CFA, type strength can be estimated by $\hat{\tau }=(n_{\mathrm{ijkl}}/e_{\mathrm{ijkl}})‐1$. In Victor’s CFA, the estimator is $\hat{\tau }=(n_{\mathrm{ijkl}}/V_{\mathrm{ijkl}})‐1$, where ê_ijkl_ and *V_ijkl_* are the estimated expected frequencies.

For an example, consider the sample cross-classification in Table 1, in which three Victor types where identified. For these types, we obtain the type strength estimates ${\hat{\tau }}_{1}=(15/6.99)\;-\;1\;=\;1.14$, ${\hat{\tau }}_{2}=(38/7.32)\;-\;1\;=\;4.19$, and ${\hat{\tau }}_{3}=(34/9.66)\;=\;2.52$, with an average of $\hat{\tau }=2.62$. this value is close to the a priori set value of type strength of τ = 2.5. The number *j* of the combinatorial Victor type cells is, in the combinatorial search, a known parameter.

In the following simulation, we estimate the values of *k* that need to be set to conserve the global level *α_G_* = 0.05. The simulation was conducted in 2 × 3 runs under variation of *n*_ijkl_, τ, and *j*. Parameter *j* assumed the values 0, 1, 2, 3, and 4. The algorithm involves the following three steps.

1. A minimum number, *n_f,min_*, is apriori determined for the number of incorrectly identified types. This number is given by *n_f,min_* =0; if ((rand()/RAND_MAX) < 0.05), n_f,min_ =1. Based on this definition, the minimum assumes a value of zero with probability 95%, and the value of 1 with probability 5%.
2. For each table, the number *n_f,g_* of incorrectly identified types is determined and parameter *k* is set to zero.
3. When *n_f,g_* > *n_f,min_*, a cycle is started for *k* = 1, 2, 3, …, in which, for the newly protected α, that is, for *α_k_* = *f_k_* α*, where α* is the Bonferroni-protected α, the value of *n_f,g_* is determined up until a value results in *n_f,g_* ≤ *n_f,min_*. This value of *k* is retained.

To vary type strength, the four type cells (4, 12, 19, and 22 out of the 24 cells of the sample table) were examined under four strength levels: τ =(0.5, 1.0, 1.5. 2.0) for τ=1.25, (1.5, 2.0, 2.5. 3.0) for τ=2.25, and (2.5, 3.0, 3.5. 4.0) for τ =3.25. Table 4 shows the results of this simulation with respect to the average cell frequency, *n*_ijkl_, the average type strength, τ, and the number, *j*, of combinatorial Victor type cells, with *j* = 0, 1, 2, 3, 4. The values of *k* are averaged over the four type cells and 200 simulation runs. Table 4 summarizes results.

**Table 4:** k, α_G_, and 1 – β values under variation of the average cell frequency, n_ijkl_, the average type strength, τ, and the number j of combined Victor type cells.

*n*_ijkl_	τ		j=0	j=1	j=2	j=3	j=4
10	1.25	*k*	0	0	2	6	8
		*α_G_*	0.00	0.00	0.00	0.01	0.03
		*1-β*	0.32	0.32	0.31	0.32	0.32
10	2.25	*k*	1	0	3	8	13
		*α_G_*	0.01	0.00	0.01	0.01	0.01
		*1-β*	0.46	0.46	0.45	0.44	0.46
10	3.35	*k*	6	3	9	13	7
		*α_G_*	0.01	0.01	0.02	0.00	0.01
		*1-β*	0.51	0.51	0.48	0.45	0.45
20	1.25	*k*	3	1	2	3	10
		*α_G_*	0.00	0.00	0.00	0.01	0.01
		*1-β*	0.39	0.38	0.38	0.38	0.39
20	2.25	*k*	18	4	2	4	6
		*α_G_*	0.05	0.00	0.01	0.00	0.00
		*1-β*	0.66	0.66	0.66	0.64	0.64
20	3.25	*k*	52	26	11	4	1
		*α_G_*	0.05	0.02	0.01	0.00	0.00
		*1-β*	0.74	0.76	0.73	0.72	0.72

The results in Table 4 suggest that, overall, the global level *α_G_* was sufficiently protected. However, the additional adjustment resulted in a loss in power of the magnitude of *Δ(*1 *- β*).This loss was calculated as the difference *Δ(*1 *- β)= (*1 *- β)_with_ – (*1 *- β)_without_* between the power *with* and *without* the additional adjustment. Table 5 summarizes the loss in power.

**Table 5:** Loss in power, Δ(1-β) = (1-β)_with_ - (1-β)_without_, from the additional adjustment of α_G_.

*n*_ijk_	τ	*n*_ijkl_	j=0	j=1	j=2	j=3	j=4
10	1.25	Δ(1-β)	+0.18	+0.03	-0.09	-0.13	-0.13
10	2.25	Δ(1-β)	+0.03	+0.02	-0.06	-0.19	-0.24
10	3.25	Δ(1-β)	-0.20	-0.11	-0.14	-0.24	-0.39
20	1.25	Δ(1-β)	-0.03	-0.08	-0.13	-0.20	-0.21
20	2.25	Δ(1-β)	-0.10	-0.05	-0.06	-0.15	-0.26
20	3.25	Δ(1-β)	-0.17	-0.09	-0.12	-0.19	-0.24

Column *j* = 0 contains the results for standard CFA, that is, for a CFA without Victor type cells. Here, when type strength is low, loss in power is unproblematic, it hovers about zero. In fact, power may increase, as is indicated by positive values (shaded green). In contrast, a combinatorial search can result in loss of power and is, therefore, counterproductive (Columns *j* = 1 through *j* = 4). A different picture emerges for greater type strengths (*τ*=2.25 and 3.25). When at least one Victor type is considered, losses in power are minimal.

We conclude that, in the attempt to minimize the global level error, *α_G_*, while maximizing power, 1 - β, it can be useful to focus on *α_G_*. The reason for this is that a reduction in *α_G_* always results in an increase in β and, thus, in a reduction of power, 1 – β.

## Combinatorial Search With *α – β* Screening

The results presented in the last section suggest that the identification of types and antitypes depends on the a priori specified error α. Now, in fuzzy logic (Zadeh, 1965), not just the truth values *true* and *false* are used, but any value in between as well. In other words, the entire interval [0, 1] is available. With reference to CFA, we can discuss the existence of types and antitypes, but also the probability, *P_β_ =1 - β*, that a type or antitype surfaces. That is, we can also discuss the power of the test against the null hypothesis of no type or no antitype.

This power, again, depends on the a priori specified error *α*. Therefore, and with reference to fuzzy logic, it can be interesting to simulate the function *P_β_ (α)* for each of the cells of a table. The data analyst can, then, based on the resulting curves, derive statements concerning the power with which types and antitypes emerge, depending on the a priori specified error *α*.

In the present section, we present a simulation of the analysis of a frequency table with *α – β* screening. This simulation involves the following three steps:

1. Conducting a Victor CFA; the number of combined cells is *n_t,max_*.
2. Depending on the global level *α_G_*, *n_s_*_,_*_t_* = INT(1/*α_G_*) cross-classifications are simulated. This number is, for *α_G_* = 0.05, *n_s_*_,_*_t_* = INT(1/*α_G_*) = 20. In the simulation, only cell frequencies are stochastically varied.
3. To represent simulation results, the local level error is continuously lowered from its start value, *α_0_ = α_G_* = 0.05, using the formula *α_k_= α_0_* 2^-^*^k^* for *k*=1, 2, 3, … For each α*_k_*, it is tested whether a cell constitutes a type or an antitype. When, for parameter *k*, Cell *j* constitutes *n_i,k_* times out of the *n_s,t_* simulated tables a type or an antitype, the estimator of type probability, *P_β_*, is given by *P_β_* = *n_i,k_* / *n_s,t_*. For any given cell, a graph of the trajectory of *P_β_* can, then, be created. This is exemplified in the examples in the next section.

## Results

In this section, we present two real-world data examples, in which we apply the approach proposed in the last section.

*Example 1: Smoking habits*. In the first example, we use data from a 2-year study of smoking behavior of German and Indian students. The participants were asked during lectures on biomedical statistics. The following variables were used in the example:

- Gender (G; *f* = 1; *m* = 2),
- Citizenship (C; India = 1; Germany = 2),
- Year (Y; 2017 = 1; 2018 = 2), and
- Smoking habit (S; does not smoke = 1; smoker = 2).

Table 6 contains the observed frequencies for the cross-classification of these four variables. Table 7 displays the results of Victor CFA.

**Table 6:** Cross-classification of Gender, Citizenship, Year of Observation, and Smoking Habit.

Cell number	G	C	Y	S	Freq.
1	1	1	1	1	19
2	1	1	1	2	15
3	1	1	2	1	14
4	1	1	2	2	9
5	1	2	1	1	11
6	1	2	1	2	9
7	1	2	2	1	13
8	1	2	2	2	11
9	2	1	1	1	0
10	2	1	1	2	12
11	2	1	2	1	12
12	2	1	2	2	9
13	2	2	1	1	13
14	2	2	1	2	11
15	2	2	2	1	10
16	2	2	2	2	14

**Table 7:** Victor CFA on smoking habit with the four design variables, frequencies, Victor expectations, test values, critical error probabilities and the information type, antitype or nothing, and j=3 (3 Victor type cells in combination).

Cell number	G	C	Y	S	Fr	Ve	Tv	CEP	T/A
1	1	1	1	1	**19**	16.99	0.47	0.64	
2	1	1	1	2	**15**	12.67	0.63	0.53	
3	1	1	2	1	**14**	13.20	0.21	0.83	
4	1	1	2	2	**9**	9.92	-0.28	0.78	
5	1	2	1	1	**11**	13.20	-0.58	0.56	
6	1	2	1	2	**9**	0.92	-0.28	0.78	
7	1	2	2	1	**13**	10.32	0.79	0.43	
8	1	2	2	2	**11**	7.84	1.06	0.29	
**9**	**2**	**1**	**1**	**1**	**0**	**15.84**	**-3.86**	**0.0001**	**At**
10	2	1	1	2	**12**	11.83	0.05	0.96	
11	2	1	2	1	**12**	12.33	-0.09	0.93	
12	2	1	2	2	**9**	9.28	-0.09	0.93	
13	2	2	1	1	**13**	12.33	0.18	0.85	
14	2	2	1	2	**11**	9.28	0.53	0.59	
15	2	2	2	1	**10**	9.66	0.10	0.92	
16	2	2	2	2	**14**	7.37	2.28	0.023	

**Figure 1:**
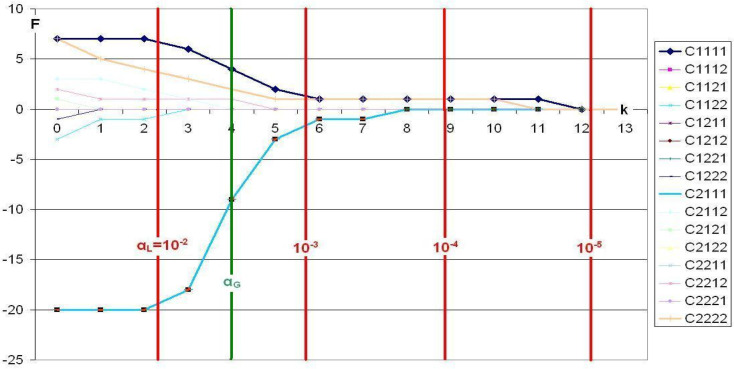
Smoking habit, analyzed using standard CFA, α_G_=0.05.

**Figure 2:**
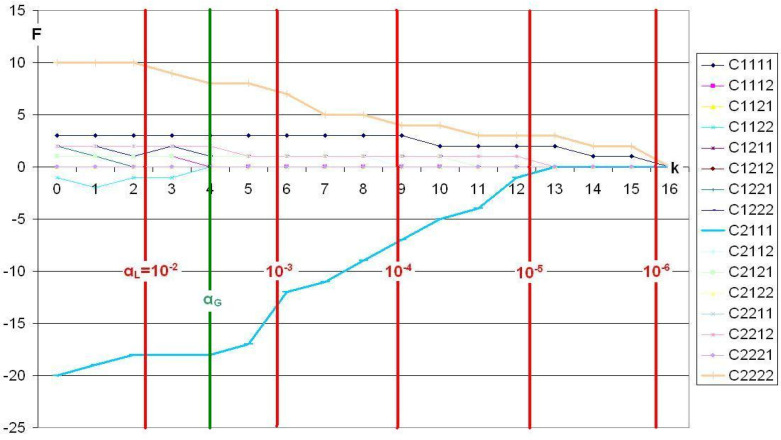
Smoking habit, analyzed using Victor CFA, α_G_=0.05.

In the following graphs, trajectories are depicted first for standard CFA (Figure 1) and, then, for Victor CFA (Figure 2). The trajectories are cell-specific. Parameter *k* – it controls the local level *α* – is represented by the abscissa. The ordinate gives the frequency of a particular cell constituting a type or antitype. As *k* increases, the local level α decreases and, thus, the number of times a cell is found to constitute a type or antitype. Cells that constitute antitypes are represented by negative frequencies. Power, *P_β_* (*k*), is directly related to type/antitype frequency, *P_β_* (*k*) = F(*k*)/*n_s_*_,t_. The vertical red lines mark the powers of ten of α_L_, while the vertical green α_G_-line represents the α_L_ at the given α_G_ = 0.05 with α_L_ = α_G_/16 (Bonferroni adjustment for 16 cells).

**Figure 3:**
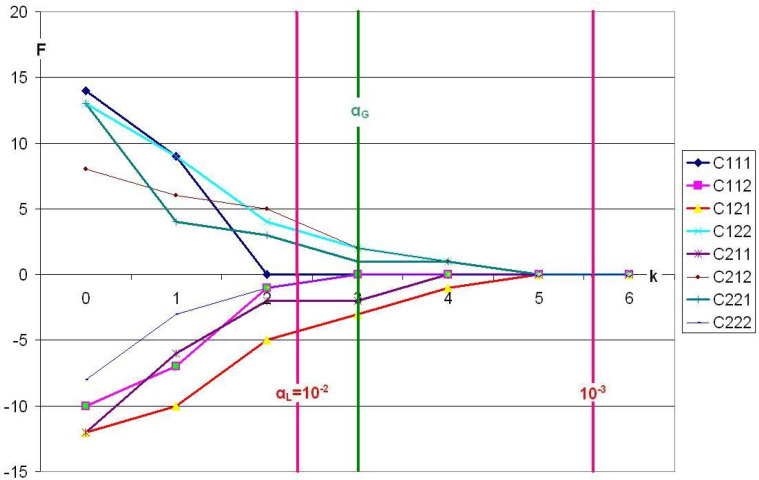
Lienert’s LSD data, analyzed using standard CFA, with α_G_=0.05.

**Figure 4:**
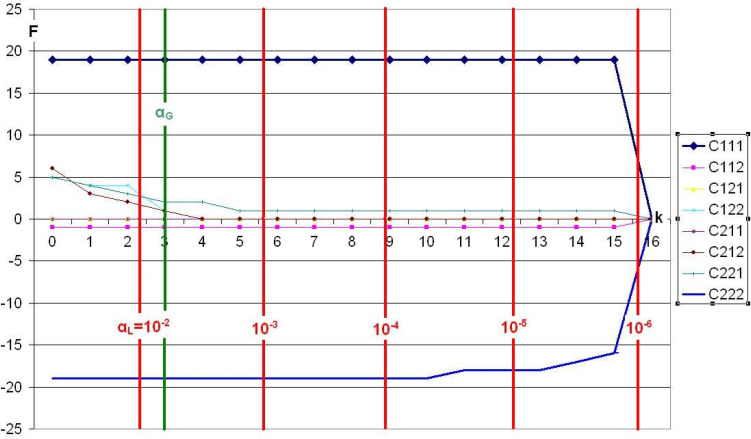
Lienert’s LSD data, analyzed using Victor CFA, α_G_=0.05.

The results in Figure 1 suggest that, the type that is constituted by Cell 1 1 1 1 (black line) is comparatively weak. For *k* = 0, that is, *α_L_* = 0.05, this cell suggests the presence of a type in only seven out of the 20 simulated instances (Power = 7/20 = 0.35). As *k* increases, this frequency sinks to a value of 1. Beginning with *k* = 12, that is, for *α_L_*~10 ^-5^, this type disappears entirely. Similarly, Cell 2 2 2 2 (yellow line), also constitutes a comparatively weak type. Its first type frequency is five, and it approaches zero even more rapidly than Cell 1 1 1 1. In contrast, Cell 2 1 1 1 (turquoise line) constitutes a comparatively strong antitype. It starts from an antitype frequency of 20. However, with increasing *k*, it also approaches zero. More specifically, beginning with *k* = 8, that is, beginning with *α_L_* ˜ 10 ^-4^, this antitype has completely disappeared.

When Victor’s CFA is applied, Cell 2 2 2 2 (brown line) constitutes a type of average strength. For *k* = 0, that is, *α_L_* = 0.05, this cell suggests the presence of a type in *f* = 10 out of 20 instances (Power = 10/20 = 0.5). As *k* increases, that is, as *α_L_* decreases, this frequency decreases as well. Still, for *α_L_*~10 ^-4^, type frequency remains at *f* = 5 (Power = 0.25). This type does not disappear before *k* > 16, that is, *α_L_*~10 ^-4^. Cell 2 1 1 1 (Row 9; turquoise line) constitutes a strong antitype, just as in standard CFA. For *k* = 0, that is, *α_L_* = 0.05, it is identified ten times as constituting an antitype. Yet, here again, power *P_β_* (*k*) decreases as *k* increases. Beginning with *α_L_*~10 ^-4^, this frequency is reduced to *f* = 4 (Power = 0.20), and for *α_L_*~10 ^-5^, it approaches zero.

***Example 2: Leuner’s LSD data*.** In the second example, we re-analyze Leuner’s (1962) LSD data, that is, Lienert’s (1968) original CFA data. In Leuner’s study, students took LSD, under controlled conditions. The participants were asked whether they experienced Narrowed Consciousness (C), Thought Disturbances (T), or Affect Disturbances (A). These three variables were dichotomized so that a 1 suggests above average and a 2 suggests below average. Table 8 shows the C × T × A cross-classification including the observed cell frequencies.

**Table 8:** Standard CFA analyzed using Lienert’s LSD data with the three design variables, observed cell frequencies, expected cell frequencies E, test values Tv, critical error probabilities CEP, and the information type, antitype or nothing, j=0 (No Victor types assumed).

Cell number	C	T	A	Fr	E	Tv	CEP	T/A
1	1	1	1	20	12.51	2.04	0.042	
2	1	1	2	1	6.85	-2.08	0.038	
3	1	2	1	4	11.40	-2.10	0.036	
4	1	2	2	12	6.24	2.13	0.034	
5	2	1	1	3	9.46	-1.99	0.046	
6	2	1	2	10	5.18	1.92	0.055	
7	2	2	1	15	8.63	2.05	0.041	
8	2	2	2	0	4.73	-1.95	0.051	

The 8 cells contain 4 types and 4 antitypes. However, at the green α_G_-line, the frequency of all 8 cells has dropped to values *f* ≤ 3 (power ≤ 3/20=0.15). Thus, none of the cells has a real chance of being identified as type or antitype, as shown in Table 8. The CEP would have to be below the value α*=0.05/8=0.00625, which all 8 cells are far from.

The Victor CFA paints a completely different picture. Cell C111 is a strong type with a power of 0.95, maintaining its performance until termination (our C program only calculates CEP values for CEP ≥ 10^-6^). The antitype of cell C222 is not quite as strong. It begins to collapse for *α_L_* values < 10^-4^, but still has a power of 17/20 = 0.85 for *α_L_* = 10^-5^. All other cells show frequencies of *f*<2 for *α_L_*-values < 10^-3^. Cells 3 and 5 (C121 and C211) generally had frequency *f*=0 from *k*=0 onwards in the simulation, so they have completely disappeared from the plot.

It is surprising that, despite the high type and antitype strength of cells 1 and 8 (C111 and C222), the frequency of 20 was not reached. A closer examination revealed that one table (Table 9 of the 20 simulated tables) was out of sequence. We do not consider this a programming error, but rather an illustration of the well-known fact that even in real data collection, outliers with a small but finite probability are possible.

**Table 9:** Victor-CFA analyzed using Lienert’s LSD data with the three design variables, observed cell frequencies, expected cell frequencies E, test values, critical error probabilities, and the information type, antitype or nothing, j=2 (2 Victor type cells in combination).

Cell number	C	T	A	Fr	Ve	Tv	CEP	T/A
1	1	1	1	20	2.00	8.78	10-6	T
2	1	1	2	1	2.39	-0.68	0.50	
3	1	2	1	4	3.07	0.45	0.65	
4	1	2	2	12	7.55	1.51	0.13	
5	2	1	1	3	2.72	0.14	0.89	
6	2	1	2	10	6.14	1.43	0.15	
7	2	2	1	15	10.22	1.43	0.15	
8	2	2	2	0	30.91	-5.47	10-6	At

## Template for Using the Program

Our guide is divided into two parts: (1) Pragmatic selection of the best CFA method. This uses only information provided by our C program in its result tables. (2) A parameter-driven analysis for further information.

For the pragmatic selection of the best CFA method, the program proposes two criteria:

1. A change in the type/antitype configuration occurs when transitioning from standard CFA (no Victor type cells hypothesized) to Victor CFA (one or more Victor type cells hypothesized), or when increasing the set number *j* of Victor type cells. The type/antitype configuration can be found in the result tables in column *T/A*. In the Lienert data example, standard CFA (j=0 Victor type cells assumed) returns an empty type/antitype configuration { }, meaning no type or antitype was diagnosed. In contrast, Victor CFA with an hypothesized Victor type cell returned the type/antitype configuration {(C111, T), (C222, At)}.
2. A significant increase in the magnitudes of the test values for diagnosed types/antitypes was observed when transitioning from standard CFA to Victor CFA, or when increasing the number of Victor type cells. An increase in the test values magnitude always implies a shift in the decay of the power function *P(αL)* towards smaller α_L_ values. For example, in Lienert’s data for Cell C111 and standard CFA, the decay of the power function begins immediately at *α_L_* = 10⁻¹, whereas in Victor CFA, the power function for cell C111 can maintain its high value up to *α_L_* = 10⁻⁶.

The pragmatic selection of the probably best result is, then, carried out in two steps, using the above criteria:

1. Decision for standard CFA or Victor CFA: Stick with standard CFA if the type/antitype configuration does not change when transitioning from the standard CFA to Victor CFA.
2. Finding the optimal number *j* of assumed Victor type cells for the case where Victor CFA has been chosen: Increase the number *j* of assumed Victor type cells as long as the test values *Tv* of the diagnosed types/antitypes increase. Thus, in the example of Lienert’s data, the absolute values of the test values of the type/antitype cells diagnosed for *j=1*, Tv_C111_ = 5.22 and Tv_C222_ = 3.07, increase to the values Tv_C111_ = 8.78 and Tv_C222_ = 5.47 for *j = 2*. It is important not to exceed the recommended maximum number n_t,max_=INT[df ^0.5^+0.01] of hypothesized Victor type cells, otherwise uncontrolled changes to the type/antitype pattern may occur, which are no longer relevant. In the example of Lienert’s data, the type/antitype configuration does not change when transitioning from *j = nt,max = 2* to *j = 3*, but the absolute values of the test values for the diagnosed type/antitype cells fall to Tv_C111_ = 8.58 and Tv_C222_ = 5.10, while, at the same time, the test values of undiagnosed cells increase, e.g., from Tv_C212_ = 1.43 to Tv_C212_ = 2.16. This is an indication of overtaxing the Victor CFA.

Our C program is designed to iterate through all values of *j =* 0, 1, *…, n_t, max_*. This, along with the rules mentioned above, allows the user to decide whether standard CFA or Victor CFA is the better choice, and, in the case of Victor CFA, which number of hypothesized Victor type cells *j* should be used.

Once the pragmatic selection of the CFA method has been made, reliable estimates of the type strengths τ can be obtained, thus enabling a parameter-driven analysis for further information for interested users. The parameters *df* (degrees of freedom) and *n*_ijkl_ (mean frequency) are easy to determine. The type strengths τ are calculated using the formula *τ_i_ = ( n_i_ / ê_i_ ) - 1*. The cell frequency *n_i_* and the estimated expected value *ê_i_* can be found in columns *Fr* and *E* of the relevant results table. The relevant results table for the selected standard CFA is the one for the value *j = 0* (no Victor type cells hypothesized), and for the selected Victor CFA, the one for the selected parameter value *j* (j > 0). Type strengths of antitypes are not calculated. When several types *τ_1_, τ_2_, …, τ_n_* are diagnosed, the arithmetic mean τ is calculated. Now, all parameters are known, and the desired additional information can be found in Table 10.

**Table 10:** Situation, decision and explanation dependent on the parameters df, n_ijkl_, and τ.

	Situation	Decision	Explanation
1	sample size small	use standard CFA	Victor CFA has less power
2	df small	use standard CFA	Victorr CFA requires more *df*
3	mean frequency *n*_ijkl_= 20, type strength τ < 1.5, α protected	consider using different method of analysis	global significance threshold of 0.05 is not guaranteed
4	mean frequency *n*_ijkl_= 10, type strength 1.5 ≤ *τ* ≤ 3.0, α protected	consider using different method of analysis	power is 20% or less
5	mean frequency *n*_ijkl_= 10, type strength 1.5 ≤ *τ* ≤ 3.0, α protected	standard CFA is the method of choice	standard CFA guarantees protection of global α and has high power
6	mean frequency *n*_ijkl_= 10, type strength *τ* > 3.0, α protected	Victor CFA is the method of choice	Victor CFA guarantees protection of global α and has high power
7	mean frequency *n*_ijkl_= 20, type strength 1.5 ≤ *τ* ≤ 2.0, α protected	standard CFA is the method of choice	standard CFA guarantees protection of global α and has high power
8	mean frequency *n*_ijkl_= 20, type strength *τ* > 2.0, α protected	Victor CFA is the method of choice	Victor CFA has more power
9	additional protection of α is applied, type strength is low	standard CFA is the method of choice	no loss of power, may be even an increase; Victor CFA suffers from loss of power
10	additional protection of α is applied, type strength is higher (τ≥ 2.0)	both approaches to CFA can be used	no loss of power

The example of ,Lienert’s data has the parameters df = 4, *n*_ijkl_= 8, and τ = (20 /2.00) -1 = 9. We find in row 6 (mean frequency *n*_ijkl_= 10, type strength *τ* > 3.0, α protected) the decision “Victor CFA is the method of choice” and the explanation “Victor CFA guarantees protection of global α and has high power”.

## Discussion and Conclusions

These two examples demonstrate that both the combination of standard CFA and Victor CFA, as well as the simulation of tables similar to the original one, can yield information gains. The *α-β* screening provides additional insights into the individual types or antitypes.

Victor CFA is important because extremely strong types can produce so-called phantom types, which are based on biased estimates of the cell probabilities from the contingency table. On the other hand, Victor CFA also has its pitfalls. In combinatorial searches using too many assumed Victor cells, the same effect occurs as above: i.e., the estimation of the cell probabilities becomes inaccurate because too much unused information from the marginal sums is missing. The result is that completely different cells suddenly appear as types or antitypes. In our experience, the limit of n_t,max_ = INT[df ^0.5^+0.01] of combined Victor cells should not be exceeded.

Using the smoking habit data as an example, it was possible to demonstrate how individual frequency curves change drastically when transitioning from standard CFA to Victor CFA. A relatively weak type (C2222) with a frequency of *f* = 4 when crossing the green line becomes a moderately strong type with a frequency of f = 8 when crossing the green *α_G_* line. In contrast, the frequency curve of type C1111 with a frequency of *f* = 4 drops to *f* = 3 when crossing the green α_G_ line. It is thus dismissed as marginal. The frequency of the antitype C2111 (turquoise), however, increases from *f*=9 to *f*=18. It is upgraded from a moderate to a strong antitype by Victor CFA.

The combination of standard CFA and Victor CFA with *α-β* screening proves to be an even more valuable source of information in the evaluation of Lienert's LSD data. All eight cells have frequency responses in the standard CFA that do not exceed the frequency **f** = 3 when crossing the green α_G_ line (power = 0.15). This means that, consequently, no type or antitype was correctly identified. Only the Victor CFA reveals that cell C111 (black curve) is a strong type and cell C222 (dark blue curve) is a strong antitype. This result was also published by Victor (Kieser & Victor, 1999], who, however, used log-linear modeling instead of CFA.

A key parameter of the Victor CFA is the number *j* of assumed Victor cells. Our estimate of *j* as j = n_t,max_ = INT[df ^0.5^+0.01] combined Victor cells has proven reliable in all simulations, but has not yet been derived theoretically. Deviations from this estimate are only possible for smaller values of *j*. The simulation program offers the possibility of running *j* through all values of *j* = 0, 1, …, n_t,max_. However, even here, apart from the analyst's common sense, there is no set of rules that selects the correct value with 100% certainty. A residual uncertainty always remains.

## Action editor

Lars-Gunnar Lundh served as action editor for this article.

## Open access

This article is distributed under the terms of the Creative Commons Attribution 4.0 International License (http://creativecommons.org/licenses/by/4.0/), which permits unrestricted use, distribution, and reproduction in any medium, provided you give appropriate credit to the original author(s) and the source, provide a link to the Creative Commons license, and indicate if changes were made.
